# Mass Spectrometry-Based Untargeted Metabolomics Identifies Distinct Metabolic Signatures in Infertility: A Comparative Analysis of PCOS, POR, and NOR

**DOI:** 10.1007/s43032-025-01908-5

**Published:** 2025-06-19

**Authors:** Metin Demirel, Mehtap Alim, Fatmanur Koktasoglu, Nil Atakul, Ebru Guner, Ayse Nur Işık Aydın, Sanem Naz Kafali, Yildız Atamer, Sahabettin Selek

**Affiliations:** 1 Department of Medical Biochemistry, Bezmialem Vakif University Faculty of Medicine, 34093 Istanbul, Turkey; 2 Health Sciences Institute, Bezmialem Vakif University, Istanbul, Turkey; 3 Department of Gynaecology and Obstetrics, Istanbul Education Research Hospital, 34093 Istanbul, Turkey; 4 Üsküdar American Academy, Istanbul, Turkey; 5 Department of Medical Biochemistry, Faculty of Medicine, Beykent University, 34093 Istanbul, Turkey

**Keywords:** Untargeted metabolomics, Follicular fluid, Metabolic pathways, Infertility, Polycystic ovary syndrome, Poor ovarian reserve, Taurocholate, Trehalose-6-phosphate

## Abstract

**Background:**

Infertility affects approximately 15% of reproductive-age couples, with polycystic ovary syndrome and poor ovarian reserve being major contributing factors. Metabolomic profiling of follicular fluid offers insights into the underlying metabolic disturbances associated with these infertility phenotypes. This study aims to identify metabolic biomarkers distinguishing PCOS, POR, and male factor infertility, which may facilitate improved diagnostic and therapeutic strategies.

**Methods:**

A total of 119 participants were categorized into three groups: PCOS (*n* = 39), POR (*n* = 40), and NOR (*n* = 40). Liquid chromatography–high-resolution mass spectrometry was used for untargeted metabolomic profiling. Metabolites were identified using HMDB, MassBank, and MoNA, while pathway analysis was performed using KEGG. Statistical analyses were conducted using R and Python, including one-way ANOVA, t-tests, and Mann-Whitney U tests, with False Discovery Rate correction applied.

**Results:**

Distinct metabolic alterations were observed among the groups. Trehalose-6-phosphate, taurocholate, and N,N-dimethylglycine emerged as the most significantly altered metabolites, showing strong discriminatory potential between PCOS and POR. PCOS patients exhibited reduced levels of taurocholate, mycalemide, and trehalose-6-phosphate, whereas NOR patients showed elevated levels of N,N-dimethylglycine and argininosuccinate. The POR group demonstrated increased levels of 1-methyl-2-pyrrolidone and haplopine, along with a broader metabolite distribution.

**Conclusion:**

This study reveals phenotype-specific metabolic signatures in PCOS and POR, identifying taurocholate, mycalemide, and N,N-dimethylglycine as potential follicular biomarkers. These findings contribute to a deeper understanding of the metabolic basis of infertility and highlight the potential of follicular fluid metabolomics for precision medicine in reproductive health.

## Introduction

Infertility is defined as the inability to achieve or maintain a pregnancy despite one year of regular, unprotected sexual intercourse. According to recent estimates from the World Health Organization (WHO), infertility affects approximately 15% of couples of reproductive age and has emerged as a global health concern driven by environmental factors, psychosocial stressors, and lifestyle changes [1].

Polycystic ovary syndrome (PCOS) is one of the most common endocrine disorders among women of reproductive age, characterized by hyperandrogenism, ovulatory dysfunction, and polycystic ovarian morphology. Insulin resistance, hyperinsulinemia, chronic inflammation, and hormonal imbalances are frequently observed in PCOS and may affect the follicular microenvironment, leading to a decline in oocyte quality [2, 3].

In contrast, poor ovarian response (POR) refers to an inadequate follicular response to gonadotropin stimulation and is typically diagnosed based on the Bologna criteria, which include advanced maternal age, prior suboptimal response to stimulation, and abnormal ovarian reserve markers such as low antral follicle count (AFC) and reduced anti-Müllerian hormone (AMH) levels [4]. POR remains a significant challenge in assisted reproductive technology (ART), often resulting in reduced oocyte yield and poor clinical outcomes [5].

Although PCOS and POR lie on opposite ends of the ovarian function spectrum, both conditions are associated with alterations in the follicular microenvironment. These changes are reflected in the biochemical composition of follicular fluid (FF), which serves as a window into the metabolic status of the oocyte and surrounding granulosa cells. Previous studies have reported disruptions in purine metabolism and glycolysis in PCOS [6], while recent metabolomic investigations have identified changes in lipid metabolism, amino acid profiles, and steroid biosynthesis in POR [7]. These findings suggest that condition-specific metabolic signatures in FF may contribute to impaired oocyte competence and adverse IVF outcomes.

Despite growing interest in the metabolic basis of infertility, the precise functional implications of these alterations remain poorly understood. Most existing studies have focused on limited metabolite groups, highlighting the need for broader, untargeted investigations. Therefore, we conducted a comprehensive metabolomic analysis of FF using liquid chromatography–mass spectrometry (LC-MS/MS) in women diagnosed with PCOS or POR. A control group comprising normo-ovulatory infertile women with male or tubal factor infertility was included for comparative analysis. The primary objectives were to identify phenotype-specific metabolic signatures, elucidate underlying biochemical alterations in the follicular milieu, and explore potential biomarker candidates to support personalized diagnostic and therapeutic strategies in reproductive medicine.

## Materials and Methods

### Study Design

This study was approved by the Research Ethics Committee of Bezmialem Vakıf University (No: 173773). Women aged 20–48 years who presented to the Obstetrics and Gynecology Outpatient Clinic of Istanbul Training and Research Hospital and met the infertility criteria defined by the European Society of Human Reproduction and Embryology (ESHRE) and the American Society for Reproductive Medicine (ASRM) were included in the study [3]. Infertility was defined as the inability to conceive despite at least one year of unprotected sexual intercourse. Participants were categorized into three groups: 39 patients diagnosed with PCOS, 40 patients diagnosed with POR, and 40 control patients with normal ovarian reserve (NOR) whose infertility was attributed to male or tubal factors.

Exclusion criteria included congenital uterine anomalies, history of ovarian surgery, endometrioma, gynecologic tumors, thyroid dysfunction, hyperprolactinemia, Cushing’s syndrome, shift work, and any systemic illness or medication that could affect follicular physiology. Patients who had used hormonal contraception in the last 3 months were also excluded. All FF samples were collected during oocyte retrieval and stored at −86°C until metabolomic analysis.

### Diagnostic Criteria and Clinical Evaluation

All participants underwent routine gynecological examinations, transvaginal ultrasonography, and ovarian reserve assessment. PCOS was diagnosed according to the Rotterdam criteria requiring at least two of the following: oligo/anovulation, hyperandrogenism, or polycystic ovarian morphology on ultrasound. POR was defined by the presence of at least two of the following Bologna criteria [4], which required the presence of at least two of the following factors: advanced maternal age (≥40 years) or the presence of other risk factors for reduced ovarian reserve, including genetic, autoimmune, surgical history, previous chemotherapy or radiotherapy, or severe endometriosis; a history of poor ovarian response, defined as the retrieval of three or fewer oocytes following a conventional stimulation protocol; or abnormal ovarian reserve test results, such as an AFC of ≤5–7 or an AMH level of ≤0.5–1.1 ng/mL. Ovulation induction was initiated on day 3 of the menstrual cycle using letrozole (5 mg/day for 5 days) followed by daily recombinant FSH injections. When leading follicles reached ≥14 mm, GnRH antagonist (cetrorelix 0.25 mg/day) started. When at least two follicles measured 16–18 mm or serum estradiol exceeded 200 pg/mL per follicle, recombinant hCG was administered. Oocyte pick-up was performed 34–36 hours later under sedation and ultrasound guidance using a 17G needle. After oocyte retrieval, leftover FF was collected by embryology personnel, centrifuged at 3000 rpm for 20 minutes, and the supernatant was aliquoted (1.5–2 mL) into Eppendorf tubes. Samples were stored at −86°C until analysis.

### Metabolomic Analysis

In the analysis, thawed FF samples were used. For protein precipitation, 300 μL of FF was transferred into a separate Eppendorf tube, followed by the addition of 600 μL of methanol. The mixture was centrifuged at 10,000 g for 15 minutes to precipitate proteins. After centrifugation, the supernatant was collected, filtered through a 0.22-μm cellulose acetate filter, and transferred into HPLC vials, preparing the samples for analysis. Metabolomic analysis was conducted using LC-MS/MS to perform untargeted metabolite profiling. Experiments were conducted at Bezmialem Vakıf University Drug Research Center (İLMER) using a Q Exactive™ Plus Hybrid Quadrupole-Orbitrap™ mass spectrometer (Thermo Fisher, Germany). Chromatographic separation was achieved on a Fortis C18 column (3 μm, 150 × 2 mm). The mobile phase consisted of water with 0.1% (v/v) formic acid (Mobile Phase A) and methanol (Mobile Phase B), with a total analysis time of 20 minutes. The proportion of Mobile Phase B gradually increased from 5% to 95% by the 18th minute, followed by re-equilibration to initial conditions. Data acquisition was performed in full scan mode using positive and negative electrospray ionization.

### Raw Data Processing and Bioinformatics Analysis

Raw mass spectrometry data were converted to mzXML and mgf formats using ProteoWizard open-source software [8]. Data processing and metabolite identification were performed using MS-DIAL, TidyMass, and MetaboAnalyst software [9–11]. Metabolites were annotated using databases such as the Human Metabolome Database (HMDB), MassBank, and the MassBank of North America (MoNA), while metabolic pathway analysis was conducted using the Kyoto Encyclopedia of Genes and Genomes (KEGG).

### Statistical Analysis

Statistical analyses were performed using R-Project (v.4.4.1) and Python. Data analysis was conducted using the pandas, numpy, and scipy.stats libraries, while visualization was performed using seaborn and matplotlib. Data preprocessing and scaling were carried out using the sklearn.preprocessing module. Group comparisons were performed using independent samples t-tests and one-way ANOVA for parametric data, while Spearman correlation and Mann-Whitney U tests were applied for non-parametric data. Multiple testing corrections were conducted using the False Discovery Rate (FDR) method, and fold-change (FC) values were calculated. A significant threshold of (*p* < 0.05) or (FDR < 0.05) was considered statistically significant.

## Results

In this study, the FF metabolomes of the NOR, POR, and PCOS groups were analyzed using LC-MS/MS, and metabolic differences were evaluated through multivariate statistical methods. The findings revealed significant biochemical alterations in the POR and PCOS groups, with specific metabolites such as Trehalose 6-phosphate, N,N-dimethylglycine, Taurocholate, Argininosuccinate and Tryptophan playing a crucial role in this distinction.

Age, body mass index (BMI), and hormonal parameters among the NOR, POR, and PCOS groups were assessed using independent samples t-test and Mann-Whitney U test (Table 1). The POR group exhibited a significantly higher mean age compared to the NOR and PCOS groups (*p* < 0.001). No statistically significant difference was observed in BMI among the groups (*p* > 0.05).

**Table 1:** Comparison of clinical and hormonal parameters among NOR, POR, and PCOS groups

		POR (*N*= 40)	NOR (*N*=40)	PCOS (*N*=39)	PCOS vs NOR	POR vs NOR
					*p*-Value	
Age	Mean (SD)	35.4 (5.28)	31.26 (4.89)	29.24 (4.13)	.032^a^	.0001 ^a^
	Min - Max	24 (48)	20 (41)	20 (41)		
	Median (IQR)	36.0 (32.0–39.0)	32.0 (27.0–34.75)	29.0 (27.0–32.0)	.0415^b^	.0001 ^b^
BMI (kg/m2)	Mean (SD)	26.4 (4.82)	26.36 (4.21)	26.03 (4.67)	.7138 ^a^	.9671 ^a^
	Min - Max	17.0 (46.1)	18.9 (38.0)	18.0 (37.8)		
	Median (IQR)	25.7 (23.7–28.2)	26.15 (23.55–28.42)	25.5 (22.5–28.8)	.5889 ^b^	.9558 ^b^
AMH (ng/mL)	Mean (SD)	0.55 (0.32)	1.84 (0.73)	5.96 (3.02)	<.0001 ^a^	<.0001 ^a^
	Min - Max	0.04 (1.41)	1.04 (4.73)	2.15 (14.48)		
	Median (IQR)	0.53 (0.3–0.78)	1.5 (1.38–1.99)	5.22 (4.2–7.49)	<.0001 ^b^	<.0001 ^b^
FSH (U/L)	Mean (SD)	9.01 (4.89)	6.5 (2.14)	5.91 (1.52)	.126 ^a^	.0007 ^a^
	Min - Max	2.02 (33.3)	1.1 (11.56)	3.82 (11.0)		
	Median (IQR)	7.5 (6.15–11.35)	6.14 (4.98–7.97)	5.99 (4.72–6.51)	.1737 ^b^	<.001 ^b^
LH (U/L)	Mean (SD)	7.21 (3.26)	7.7 (6.63)	8.1 (4.08)	.7179 ^a^	.6385 ^a^
	Min - Max	1.18 (19.2)	2.82 (49.8)	2.14 (18.5)		
	Median (IQR)	6.86 (4.88–8.56)	6.22 (4.8–7.94)	7.0 (5.01–10.22)	.2865 ^b^	.6134 ^b^
E2 (ng/L)	Mean (SD)	58.76 (64.77)	45.63 (22.45)	48.82 (42.38)	.6536 ^a^	.1523 ^a^
	Min - Max	9.25 (474.3)	13.83 (97.7)	9.88 (278.1)		
	Median (IQR)	43.62 (32.46–59.5)	38.89 (28.28–62.09)	39.11 (30.3–51.34)	.908 ^b^	.3538 ^b^
TSH (mU/L)	Mean (SD)	1.98 (0.8)	2.04 (0.98)	2.09 (1.04)	.8086 ^a^	.7471 ^a^
	Min - Max	0.67 (4.18)	0.47 (5.45)	0.5 (4.91)		
	Median (IQR)	1.95 (1.44–2.4)	1.85 (1.35–2.56)	1.76 (1.38–2.65)	.7571 ^b^	.9215 ^b^
PRL (μg/L)	Mean (SD)	17.63 (9.18)	22.37 (14.65)	18.11 (8.23)	.0805 ^a^	.0513 ^a^
	Min - Max	6.97 (54.0)	2.85 (73.5)	8.53 (46.3)		
	Median (IQR)	15.96 (11.38–20.64)	16.7 (14.31–24.58)	16.5 (13.2–19.7)	.2132 ^b^	.0486 ^b^

AMH levels were found to be highest in the PCOS group and lowest in the POR group, with both comparisons showing statistically significant differences (*p* < 0.001). Follicle-Stimulating Hormone (FSH) levels were significantly higher in the POR group compared to the NOR and PCOS groups (*p* < 0.01). No significant differences were observed among the groups in Luteinizing Hormone (LH), estradiol (E2), thyroid-stimulating hormone (TSH), or prolactin (PRL) levels (*p* > 0.05).

Independent samples t-test results revealed significant metabolic differences among the NOR, POR, and PCOS groups (Tables 2, 3, and 4). In the POR group, N,N-dimethylglycine (FDR <0.05) and argininosuccinate (FDR <0.05) were found to be significantly lower, suggesting a potential association with nitrogen metabolism and methylation processes. Conversely, haplopine (FDR >0.05) was detected at higher levels in the POR group.

**Table 2:** Differential metabolites (NOR vs. POR) (This table presents the differentially abundant metabolites between the NOR and POR groups based on statistical comparison. Metabolites are ranked by p-value, with FDR correction applied.)

Metabolites	t-Statistic	*p*-Value	-log10(*p*-Value)	FDR
N,N-dimethylglycine	−3.7198	0.000374	3.4271	0.013466
Argininosuccinate	−3.317	0.001384	2.859	0.024903
Haplopine	2.8851	0.005058	2.296	0.060695
14-bromo-1-hydroxy-7,8-dehydrodiscorhabdin V	−2.1169	0.037459	1.4264	0.33713
N-Methyl-14-bromo-1-hydroxy-7,8-dehydrodiscorhabdin V	−1.6978	0.093527	1.0291	0.51778
3-Iodotyrosine	−1.6704	0.098839	1.0051	0.51778

**Table 3:** Differential metabolites (NOR vs. PCOS) (This table presents the differentially abundant metabolites between the NOR and PCOS groups based on statistical comparison. Metabolites are ranked by p-value, with FDR correction applied.)

Metabolites	t-Statistic	*p*-Value	-log10(*p*-Value)	FDR
Mycalemide	3.0776	0.002891	2.539	0.05727
Taurocholate	2.9096	0.004729	2.3252	0.05727
Malyngamide T	2.8966	0.004909	2.309	0.05727
Minocycline	2.5728	0.012011	1.9204	0.097525
Trehalose 6-phosphate	2.5125	0.014079	1.8514	0.097525
b-Ala-Lys	−2.3915	0.019222	1.7162	0.097525
Tryptophan	2.357	0.020965	1.6785	0.097525
Haplopine	−2.3324	0.022291	1.6519	0.097525
Oleandomycin	−2.1899	0.03156	1.5009	0.12273
N-Methylcoclaurine	1.9317	0.057075	1.2436	0.19976

**Table 4:** Differential metabolites (PCOS vs. POR) (This table presents the top 10 differentially abundant metabolites between the PCOS and POR groups based on statistical comparison. Metabolites are ranked by p-value, with FDR correction applied.)

Metabolites	t-Statistic	*p*-Value	-log10(*p*-Value)	FDR
Trehalose 6-phosphate	−3.8967	0.00020654	3.685	0.0023345
N,N-dimethylglycine	3.8303	0.00025934	3.5861	0.0023345
Taurocholate	−3.7734	0.00031465	3.5022	0.0023345
mycalemide	−3.7726	0.00031547	3.501	0.0023345
Minocycline	−3.4405	0.00094085	3.0265	0.0058019
Argininosuccinate	3.0907	0.0027794	2.556	0.014691
Tryptophan	−2.9632	0.0040492	2.3926	0.018727
Malyngamide T	−2.8128	0.006228	2.2057	0.025604
Puromycin	−2.7287	0.0078734	2.1038	0.026898
N-Methylcoclaurine	−2.7231	0.0079967	2.0971	0.026898
1-Methyl-2-pyrrolidone	−2.526	0.013589	1.8668	0.0419
Malyngamide J	−2.4673	0.015832	1.8005	0.045061
14-bromo-1-hydroxy-7,8-dehydrodiscorhabdin V	2.1495	0.034733	1.4593	0.091796
3-Iodotyrosine	2.0526	0.043508	1.3614	0.10732
Methoxylated and dihydrogenated discorhabdin C	1.8562	0.067251	1.1723	0.14989
Jamaicamide B	−1.8451	0.068867	1.162	0.14989
N-Methyl-14-bromo-1-hydroxy-7,8-dehydrodiscorhabdin V	1.735	0.086747	1.0617	0.17831

In the PCOS group, mycalemide (FDR >0.05), taurocholate (FDR >0.05), and malyngamide T (FDR >0.05) were found to be elevated, indicating a possible role in lipid metabolism and cellular stress response. Additionally, trehalose-6-phosphate (FDR >0.05) and minocycline (FDR >0.05) exhibited increased levels, while β-Ala-Lys (FDR >0.05) and haplopine (FDR >0.05) were detected at lower levels compared to other groups.

When comparing the PCOS and POR groups, trehalose-6-phosphate (FDR <0.05), taurocholate (FDR <0.05), and mycalemide (FDR <0.05) were significantly higher in the POR group, suggesting their potential involvement in energy metabolism and lipid regulation. In contrast, N,N-dimethylglycine (FDR <0.05) and argininosuccinate (FDR <0.05) were markedly elevated in the PCOS group, which may be associated with differences in methylation processes and nitrogen metabolism. Furthermore, minocycline (FDR <0.05) and tryptophan (FDR <0.05) were found at higher levels in the POR group, indicating potential links to antioxidant response and amino acid metabolism.

**Fig. 1:**
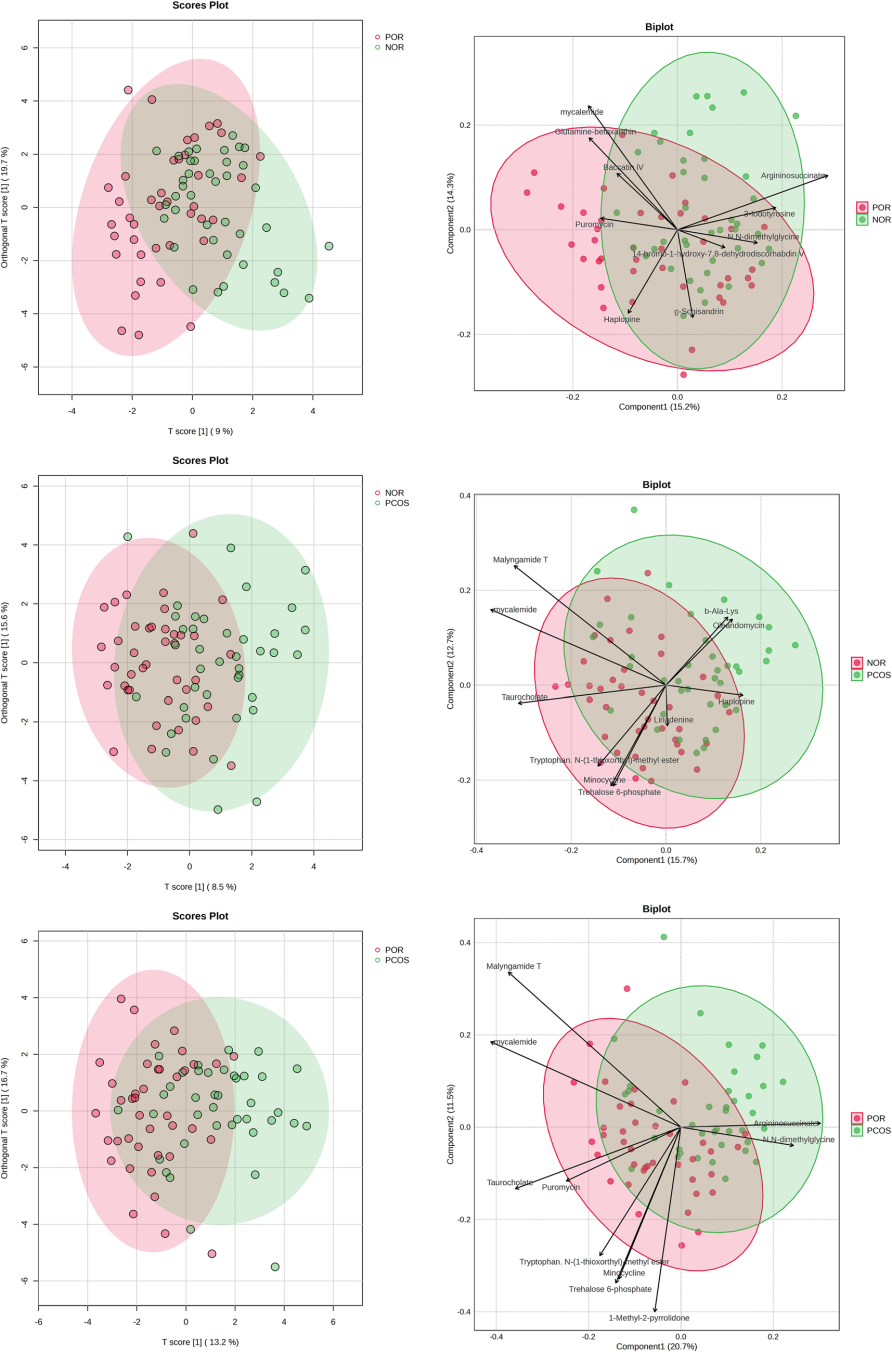
OPLS-DA and PCA analyses among NOR, POR, and PCOS groups

OPLS-DA and PCA analyses were performed to evaluate the metabolic differences among the NOR, POR, and PCOS groups (Fig. 1). The OPLS-DA score plots demonstrated a clear metabolic separation among the three groups, with POR and PCOS groups distinctly diverging from the NOR group. However, a degree of overlap among the groups suggests the presence of metabolic heterogeneity.

Biplot analyses identified specific metabolites that contributed to the differentiation of the groups. Argininosuccinate, N,N-dimethylglycine, and trehalose-6-phosphate were more prominently associated with the PCOS group, while taurocholate, mycalemide, and minocycline were predominant in the POR group.

**Fig. 2:**
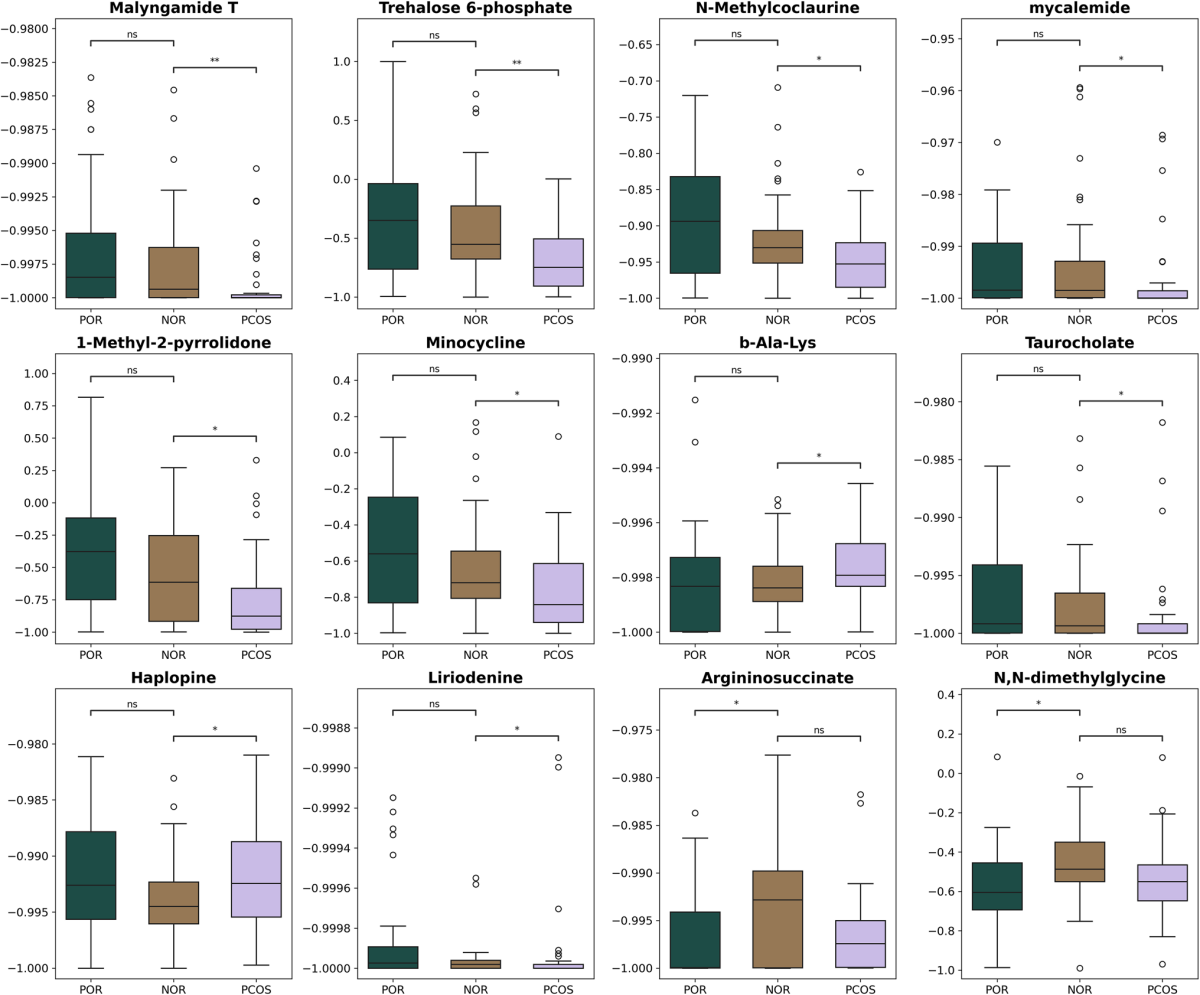
Distribution of metabolites among NOR, POR, and PCOS groups (ns: not significant; *: *p* < 0.05; **: *p* < 0.01.)

Differences in metabolite levels among the groups were visualized using box plots (Fig. 2). Trehalose-6-phosphate, taurocholate, and mycalemide were detected at the lowest levels in the PCOS group. The N,N-dimethylglycine metabolite was measured at higher levels in the NOR group compared to the other groups. Argininosuccinate levels exhibited the widest distribution in the NOR group, while being lower in the POR and PCOS groups. Minocycline and 1-methyl-2-pyrrolidone displayed a broad distribution in the POR group, whereas they were observed at lower concentrations in the PCOS group.

**Fig. 3:**
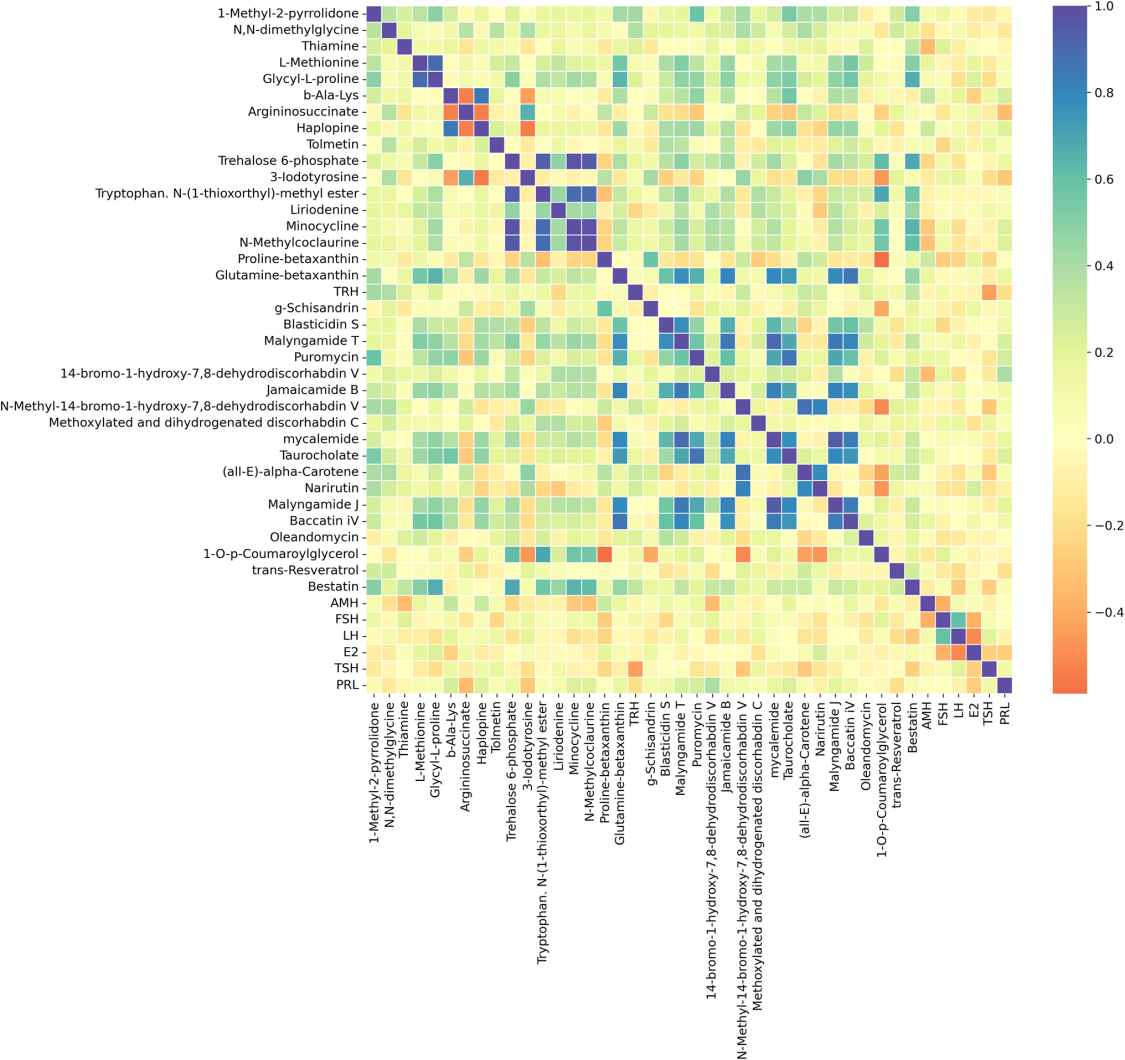
Correlation analysis of metabolites and hormones in the POR group

**Fig. 4:**
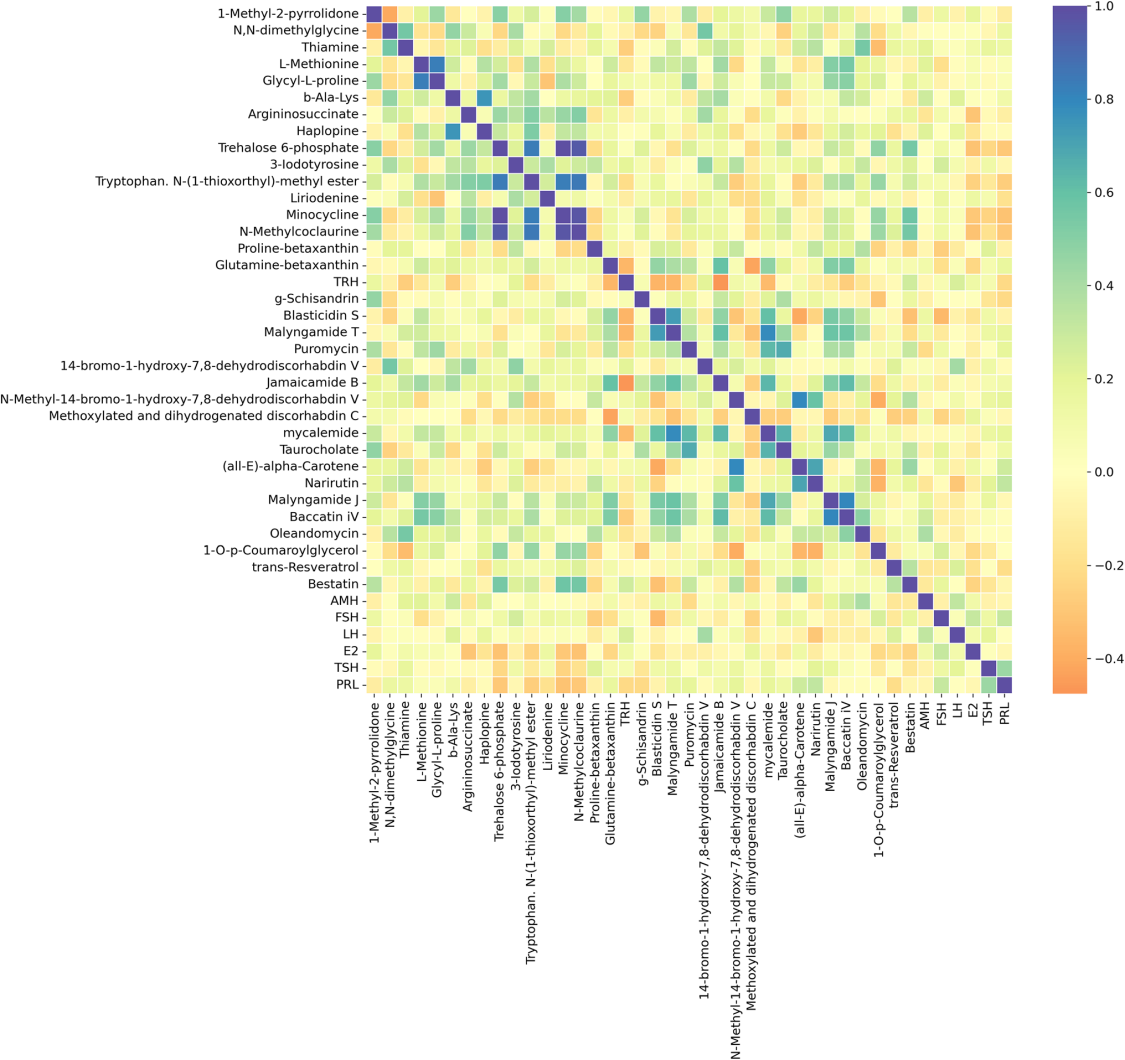
Correlation analysis of metabolites and hormones in the PCOS group

The correlation analysis conducted in the POR and PCOS groups revealed significant differences in their metabolic profiles (Figs. 3 and 4). In the POR group, L-methionine, glycyl-L-proline, and trehalose-6-phosphate exhibited strong positive correlations, suggesting coordinated regulation of amino acid metabolism and energy homeostasis. Conversely, a significant negative correlation was observed between N,N-dimethylglycine and argininosuccinate, indicating that these compounds may be associated with distinct biochemical pathways.

Regarding hormone-metabolite interactions, AMH and thiamine displayed a negative correlation, suggesting altered vitamin metabolism in individuals with poor ovarian reserves. FSH showed a weak to moderate positive correlation with trehalose-6-phosphate and E2.

In the PCOS group, a more extensive distribution of metabolic interactions was observed. 1-Methyl-2-pyrrolidone, glycyl-L-proline, and N,N-dimethylglycine exhibited strong positive correlations, indicating that these metabolites may be co-regulated within shared biochemical pathways in PCOS pathophysiology.

In terms of hormone-metabolite relationships, FSH and L-methionine demonstrated a significant negative correlation, suggesting a potential link between methionine metabolism and follicular development in PCOS. Additionally, AMH correlated weakly to moderately positively with trehalose-6-phosphate and thiamine, indicating an interaction between ovarian reserve and glucose metabolism in PCOS.

Overall, the POR group exhibited a more structured pattern of metabolic relationships concentrated around specific biochemical pathways, whereas the PCOS group displayed a broader distribution of interactions. Trehalose-6-phosphate, L-methionine, and glycyl-L-proline emerged as key metabolites in the POR group, potentially influencing poor ovarian reserve and follicular development. In contrast, 1-methyl-2-pyrrolidone, argininosuccinate, and N,N-dimethylglycine were prominent in the PCOS group, playing significant roles in cellular energy metabolism and amino acid cycling.

**Fig. 5:**
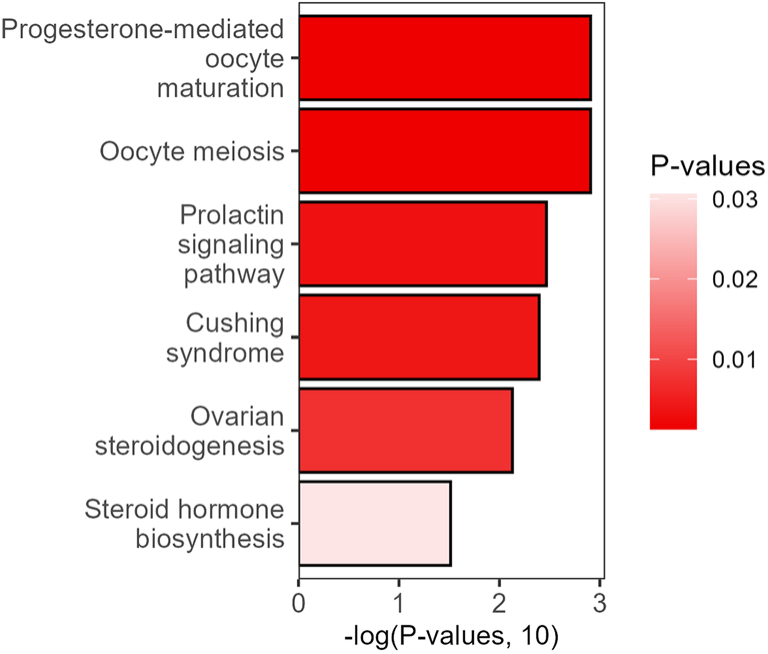
Pathway enrichment analysis of metabolic differences between PCOS and POR

Pathway enrichment analysis was conducted to evaluate the biochemical processes affected by the differential metabolites between the PCOS and POR groups (Fig. 5). The results indicate that progesterone-mediated oocyte maturation, oocyte meiosis, and the prolactin signaling pathway were significantly enriched, highlighting key metabolic distinctions between these infertility phenotypes.

Additionally, metabolic pathways associated with Cushing’s syndrome and ovarian steroidogenesis exhibited notable differences between the two groups. While steroid hormone biosynthesis displayed a relatively lower level of statistical significance, it remains a critical biochemical process reflecting the hormonal imbalances observed in PCOS and POR at the metabolic level.

**Fig. 6:**
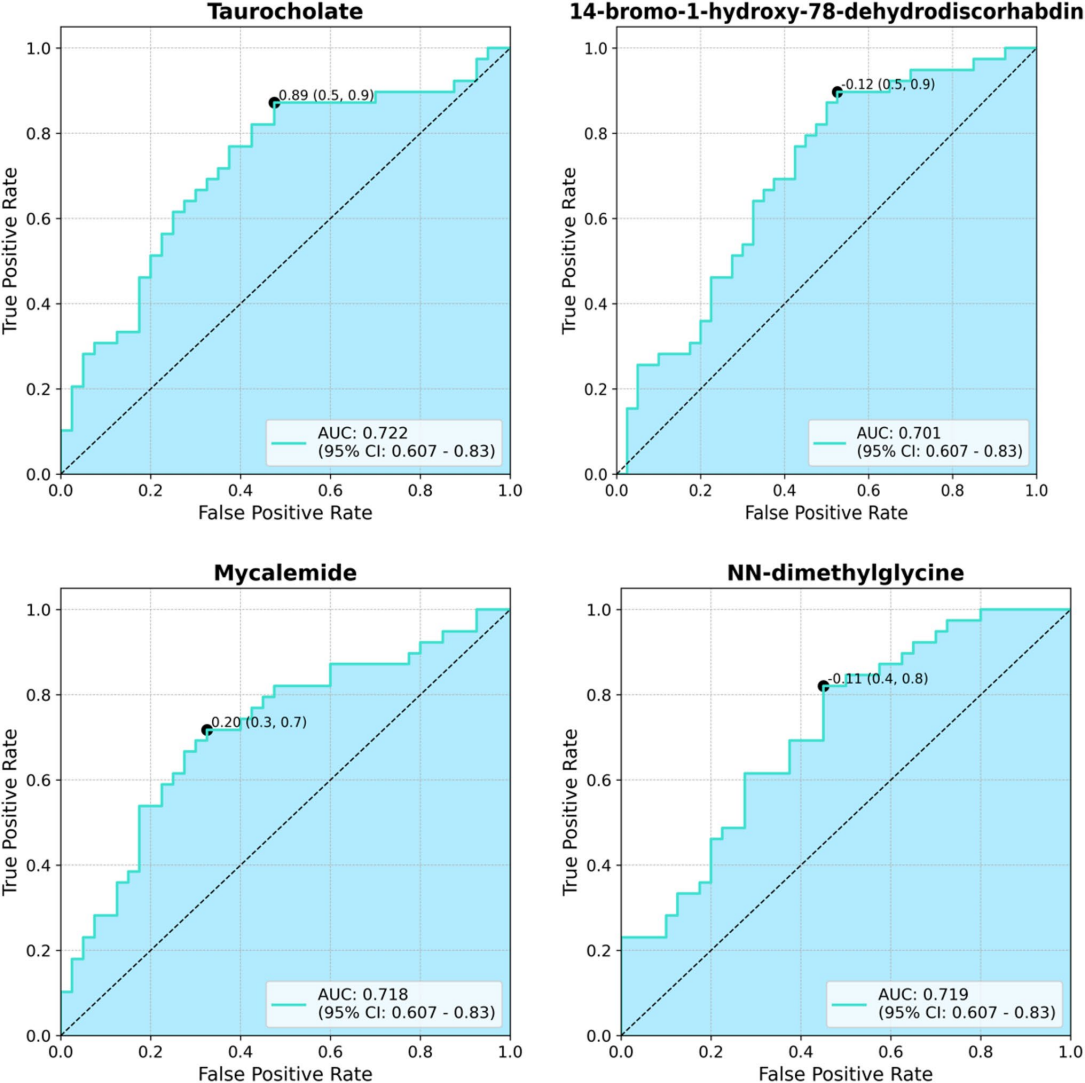
ROC analysis between POR and PCOS groups

Receiver Operating Characteristic (ROC) analysis was performed to evaluate the ability of selected metabolites to distinguish between the POR and PCOS groups (Fig. 6). The metabolites taurocholate (AUC: 0.722, 95% CI: 0.607–0.83), 14-bromo-1-hydroxy-78-dehydrodiscorhabdin (AUC: 0.701, 95% CI: 0.607–0.83), mycalemide (AUC: 0.718, 95% CI: 0.607–0.83), and N,N-dimethylglycine (AUC: 0.719, 95% CI: 0.607–0.83) demonstrated clinically significant discriminatory power in differentiating infertility phenotypes based on metabolic differences.

## Discussion

This study investigates the metabolic alterations in the FF of PCOS and POR patients, providing insights into the biochemical mechanisms underlying infertility. As a biofluid that directly reflects the local metabolic exchange between the oocyte and granulosa cells, FF provides a unique snapshot of the intrafollicular microenvironment, distinct from systemic circulation. Our findings are generally consistent with previously reported data, though some discrepancies were observed. While our metabolomic findings suggest potential biomarkers of follicular competence, functional validation through in vitro or in vivo models is necessary to confirm their mechanistic roles in oocyte quality and ovarian response.

### Carbohydrate Metabolism and Energy Production

Increased glucose accumulation and elevated TCA cycle intermediates in PCOS patients have been associated with impaired glucose transport due to insulin resistance [12–14]. In our study, the lowest levels of trehalose-6-phosphate in PCOS further support disruptions in glucose metabolism and energy homeostasis [15].

Reduced lactate levels in PCOS patients have been linked to alterations in lactate dehydrogenase (LDH) activity [16]. Additionally, the positive correlation between FSH and trehalose-6-phosphate suggests a connection between energy metabolism and hormonal regulation.

### Lipid and Hormone Metabolism

Dysregulated lipid metabolism in PCOS is evident from increased free fatty acid (FFA) levels, which may result from insulin resistance-induced failure to inhibit lipolysis, leading to FFA accumulation in the follicular microenvironment [13, 14, 17]. The lowest taurocholate levels detected in our study further suggest alterations in bile acid metabolism, which may contribute to the metabolic disturbances associated with PCOS.

In terms of hormonal regulation, the positive correlation between AMH and trehalose-6-phosphate in PCOS highlights the interplay between ovarian dysfunction and metabolic alterations. In POR, reduced 2-hydroxyestrone levels indicate hormonal imbalances that may adversely affect oocyte development [6]. Additionally, the positive correlation between FSH, trehalose-6-phosphate, and E2 suggests that hormonal fluctuations significantly influence the FF metabolic environment.

### Amino Acid Metabolism and Protein Catabolism

Increased levels of glutamate and aspartate in PCOS patients suggest that disturbances in carbohydrate metabolism may enhance protein catabolism, leading to the utilization of amino acids as alternative energy sources [12, 18].

Similarly, the broader distribution of argininosuccinate in the NOR group, along with its lower levels in the POR and PCOS groups, may reflect differences in nitrogen metabolism and amino acid utilization across these conditions. Additionally, the higher N,N-dimethylglycine levels in the NOR group suggest distinct metabolic adaptations compared to PCOS and POR.

The higher levels of L-methionine and glycyl-L-proline in POR, accompanied by lower glycine levels, may indicate increased oxidative stress and proteolysis both of which have been previously implicated in diminished ovarian function [19, 20].

### Metabolic Alterations in PCOS

Our study revealed significantly decreased levels of trehalose-6-phosphate, taurocholate, and mycalemide in the FF of PCOS patients, suggesting impaired glucose metabolism, bile acid dysregulation, and cellular stress. These findings are consistent with previous reports linking PCOS to altered energy metabolism, increased purine turnover, and accumulation of TCA cycle intermediates driven by insulin resistance [6, 12, 13, 15, 21]. The positive correlation between FSH and trehalose-6-phosphate further supports the role of gonadotropins in modulating follicular energy homeostasis.

Additionally, the reduction in lactate levels in PCOS has been linked to alterations in LDH activity [16]. Changes in LDH activity indicate a shift in cellular energy production pathways, reinforcing the hypothesis that PCOS is associated with profound metabolic adaptations.

We also observed notable lipid metabolic shifts in PCOS, including lower levels of phosphatidylcholines and higher levels of lysophospholipids, particularly lysoPCs—patterns reported in other FF-based lipidomic studies [22]. Such remodeling of membrane lipids has been associated with reduced fertilization rates and may reflect compromised oocyte quality. Additionally, bile acid profiles appear altered in PCOS; while our study showed decreased taurocholate, others have reported increases in conjugated bile acids such as GCA and GCDCA, which were positively correlated with FSH, LH, and AFC [23]. This discrepancy may reflect heterogeneity in patient characteristics or phenotype-specific differences.

Importantly, differential metabolite patterns between lean and overweight PCOS phenotypes suggest that metabolic dysfunction is not solely attributable to adiposity [24]. Even in normal-weight PCOS women, disruptions in glycerophospholipid and steroid hormone metabolism persist, along with a negative correlation between prostaglandin E2 and embryo quality, indicating intrinsic follicular abnormalities [7].

Collectively, these findings support the notion that PCOS is characterized by hormonally regulated, phenotype-specific metabolic reprogramming within the follicular microenvironment, which may contribute to compromised oocyte competence and suboptimal ART outcomes.

### Metabolic Alterations in POR

In our study, women with POR exhibited distinct metabolomic profiles compared to those with NOR and PCOS. Elevated levels of L-methionine and glycyl-L-proline in the POR group suggest increased proteolysis and the utilization of amino acids as alternative energy substrates. In contrast to PCOS patients, trehalose-6-phosphate levels were not elevated in POR but were instead lowest in the PCOS group, indicating differential regulation of glucose metabolism and energy homeostasis across infertility phenotypes.

Unlike PCOS, no statistically significant differences were observed in taurocholate or other bile acid-related metabolites in the POR group. This may reflect age-related metabolic alterations or the heterogeneity of underlying etiologies contributing to diminished ovarian reserve. Nonetheless, the identification of differentially expressed metabolites such as taurocholate, N,N-dimethylglycine, and argininosuccinate in both POR and PCOS groups aligns with previous reports highlighting the roles of bile acid biosynthesis, lipid metabolism, and methylation in ovarian dysfunction [25].

Although our study did not directly measure prostaglandins, several lipid-related metabolites, including taurocholate and malyngamide T, showed significant differences. These findings suggest a possible involvement of prostaglandin-associated biosynthetic pathways in follicular function, consistent with previous studies by Liang et al. and Song et al., which demonstrated downregulation of prostaglandin metabolites in women with diminished ovarian reserve [26, 27].

Recent studies investigating the impact of adjuvant therapies, such as growth hormone and dehydroepiandrosterone (DHEA) supplementation, have demonstrated substantial changes in FF metabolite profiles, including alterations in S-adenosylmethionine (SAM) and various fatty acid species. [28]. These observations raise the possibility that key metabolites identified in our study may not only serve as diagnostic or prognostic biomarkers but also function as pharmacometabolomic indicators, reflecting metabolic responsiveness to therapeutic interventions.

Finally, the considerable variability in POR diagnostic criteria and the absence of standardized metabolomic thresholds across studies pose challenges for clinical implementation. As emphasized in recent reviews [29], international consensus on biomarker definitions, sample timing, and data processing pipelines is critically needed to improve reproducibility and cross-study comparability. Thus, our results should be considered hypothesis-generating and underscore the importance of future multi-center studies for validation.

## Conclusion

This study provides a comprehensive metabolomic characterization of FF in women with PCOS and POR, revealing phenotype-specific biochemical signatures that may underlie infertility pathophysiology. Key metabolites such as trehalose-6-phosphate, taurocholate, and 1-methyl-2-pyrrolidone were significantly altered in PCOS, whereas N,N-dimethylglycine, argininosuccinate, and L-methionine were distinctively altered in POR. The inclusion of a NOR group enabled robust comparative analysis and enhanced the interpretability of disease-specific metabolic alterations.

A major strength of this study lies in its untargeted metabolomics approach, which enabled the broad-spectrum profiling of follicular metabolites. By integrating clinical hormonal data and correlation analyses, the study also provides insight into hormone–metabolite interactions within the follicular microenvironment. Importantly, several metabolites demonstrated moderate discriminatory capacity (AUC > 0.70) in ROC analysis, suggesting their potential utility as candidate biomarkers to differentiate between infertility phenotypes.

Nevertheless, these findings are exploratory in nature. The limited sample size and the absence of functional validation constrain the generalizability of the results. To confirm the clinical relevance of the identified biomarkers, future studies should incorporate targeted metabolomic validation within larger, multi-center cohorts. Additionally, integrating metabolomic data with complementary omics platforms may offer deeper mechanistic insights and facilitate the development of personalized therapeutic strategies in reproductive medicine.
